# Interaction between *Mycobacterium avium *subsp. *paratuberculosis *and environmental protozoa

**DOI:** 10.1186/1471-2180-6-63

**Published:** 2006-07-13

**Authors:** Lynne Whan, Irene R Grant, Michael T Rowe

**Affiliations:** 1 Department of Food Science, Queen's University Belfast, Newforge Lane, Belfast BT9 5PX, Northern Ireland, UK; 2 Food Microbiology Branch, Agriculture, Food and Environmental Science Division, Agri-Food and Biosciences Institute, Newforge Lane, Belfast BT9 5PX, Northern Ireland, UK

## Abstract

**Background:**

Interactions between *Mycobacterium avium *subsp. *paratuberculosis *(*Map*) and free-living protozoa in water are likely to occur in nature. The potential impact of ingestion of *Map *by two naturally occurring *Acanthamoeba *spp. on this pathogen's survival and chlorine resistance was investigated.

**Results:**

Between 4.6 and 9.1% of spiked populations of three *Map *strains (NCTC 8578, B2 and ATCC 19698), which had been added at a multiplicity of infection of 10:1, were ingested by *Acanthamoeba castellanii *CCAP 1501/1B and *A. polyphaga *CCAP 1501/3B during co-culture for 3 h at 25°C. *Map *cells were observed to be present within the vacuoles of the amoebae by acid-fast staining. During extended co-culture of *Map *NCTC 8578 at 25°C for 24 d with both *A. castellanii *and *A. polyphaga Map *numbers did not change significantly during the first 7 days of incubation, however a 1–1.5 log_10 _increase in *Map *numbers was observed between days 7 and 24 within both *Acanthamoeba *spp. Ingested *Map *cells were shown to be more resistant to chlorine inactivation than free *Map*. Exposure to 2 μg/ml chlorine for 30 min resulted in a log_10 _reduction of 0.94 in ingested *Map *but a log_10 _reduction of 1.73 in free *Map *(p < 0.001).

**Conclusion:**

This study demonstrated that ingestion of *Map *by and survival and multiplication of *Map *within *Acanthamoeba *spp. is possible, and that *Map *cells ingested by amoebae are more resistant to inactivation by chlorine than free *Map *cells. These findings have implications with respect to the efficacy of chlorination applied to *Map *infected surface waters.

## Background

*Mycobacterium avium *subsp. *paratuberculosis *(*Map*) is the known cause of Johne's disease of wild and domestic ruminants, in particular dairy cattle [1]. Although cattle are usually infected with *Map *during the first six months of life, via the faecal oral route, clinical signs, such as diarrhoea, emaciation and dehydration, usually develop only after a 3–5 year incubation period [2]. Despite the importance of Johne's disease, both from an animal welfare and agro-economic perspective, the molecular mechanisms that are involved in the entry and survival of *Map *in the host are only poorly characterised [3]. However, it is now generally accepted that the persistence of *Map *in host macrophages is crucial to the establishment and progression of the disease [1].

In addition to its clear veterinary importance *Map *may have a more direct human impact in that it has been implicated as a cause of Crohn's disease in humans [4]. Crohn's disease is a chronic inflammatory bowel disorder that commonly affects the terminal ileum but can occur in any part of the gastrointestinal tract from mouth to anus. At present there is no recognised cure but sufferers can experience periods of remission. However, the overall quality of life of the sufferer and their immediate family is low [5]. Although a role, if any, for *Map *as a contributory factor in Crohn's disease is not proven, the evidence was sufficient for the UK government to advocate a precautionary approach and to attempt to minimise exposure of the public to this organism [6].

*Map *is known to survive for protracted periods in the environment [7,8], although it is generally considered unable to replicate because of its requirement for mycobactin, an iron-chelating compound, required for replication of the organism outside its natural host. One of the strategies adopted by *Map *that contributes to its longevity in the environment may be its ability to survive ingestion by protozoa, which are usually bacteriovores (for a review see [9]). Indeed it has been reported that this allows *M. avium *to acquire a phenotype more pathogenic to humans [10,11]. *Legionella pneumophila*, a known human pathogen, shares the same characteristic except that it is able to multiply within its protozoan host [12]. There is a paucity of published information on the interaction of *Map *and free-living protozoa that may allow viable intervention strategies to be devised. There is also a lack of information on the protection such an intracellular location might afford against bacteriocidal agents such as chlorine that are used in water treatment operations and food plant cleaning processes.

The work reported here had three objectives: to investigate the uptake of *Map *by two commonly occurring environmental protozoa, *Acanthamoeba castellanii *and *A. polyphaga*; to determine the survival ability of *Map *when ingested by the two protozoa, and to compare the chlorine resistance of ingested and free-living *Map*.

## Results

### Ingestion of *Map *by *Acanthamoeba *spp

**Figure 1:**
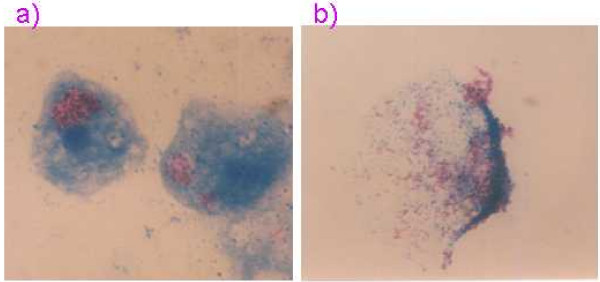
Acid-fast stained smear of (a) co-culture of *Map *and *A. castellanii *after 3 h at 25°C showing *Map *located within vacuole of amoeba cell, and (b) *Acanthamoeba *cell lysed by sonication in the presence of 1% Tween 80 releasing ingested *Map*.

Both qualitative and quantitative methods were employed to assess if ingestion of three *Map *strains by the two *Acanthamoeba *spp. occurred. Intracellularly located *Map *were initially visualised by acid-fast staining of aliquots of co-culture after 180 min; acid-fast *Map *cells staining red and subcellular structures of the amoeba staining blue. Microscopy revealed that numerous *Map *cells were present within vacuoles within the amoebae (Figure 1a). Figure 1b shows an amoeba cell lysed by sonication in the presence of 1% Tween 80 releasing the ingested *Map *cells. Enumeration of the *Map *cells ingested by the two *Acanthamoeba *spp. revealed that between 4.6–9.1% and 4.9–8.3% of the initial spiked *Map *populations were taken up by *A. castellanii *and *A. polyphaga*, respectively, with only minor differences between the three *Map *strains studied.

### Survival of *Map *within *Acanthamoeba *spp. for extended periods

**Figure 2:**
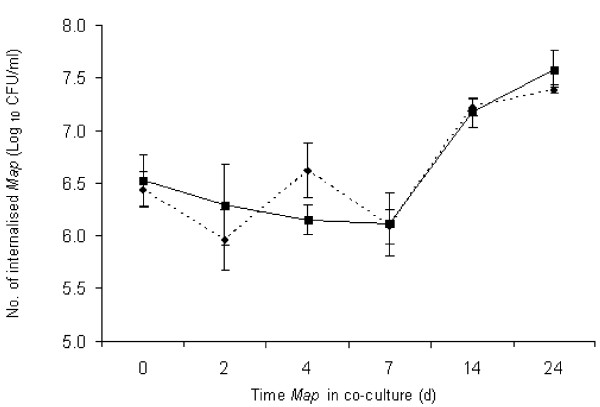
Survival of *Map *NCTC 8578 after co-culture with *Acanthamoeba castellanii *(dashed line) and *A. polyphaga *(solid line) for up to 24 days at 25°C.

When *Map *NCTC 8578 was incubated at 25°C in co-culture with either *A. castellanii *or *A. polyphaga *for a longer period (up to 24 d), the intracellular *Map *counts did not alter significantly within either *Acanthamoeba *sp. during the first 7 days of incubation (Figure 2). However, between 7 and 24 days incubation there was a significant 1–1.5 log_10 _increase in numbers of intracellular *Map *(P < 0.001). Co-culture was not followed beyond 24 d.

### Comparative chlorine resistance of free and internalised *Map*

A significant difference in the chlorine resistance of *Map *harboured within protozoa (i.e. ingested *Map*) and free *Map *was observed at all chlorine concentrations and contact times (p < 0.001). Ingested *Map *was found to be more resistant to chlorination than free *Map *overall. The greatest reduction in numbers of ingested *Map *achieved by exposure to the highest chlorine concentration (2 μg/ml) for the longest contact time (30 min) was 0.94 log_10 _reductions, whereas under the same conditions a reduction of 1.73 log_10 _was achieved for free *Map *(Table 1). Overall, chlorine concentration was found to have a significant effect on inactivation of *Map *(p < 0.05), irrespective of whether they were intracellularly located or free, whereas chlorine contact time did not have a significant effect on *Map *inactivation (p = 0.342).

## Discussion

This study has demonstrated that *Map *is not only able to survive ingestion by *A. castellanii *and *A. polyphaga*, but that the intracellular location appears to provide an environment where multiplication of *Map *can occur and protection from the effects of chlorine is afforded. Our findings in relation to ingestion capability and trends in survival of *Map *within amoeba following ingestion are similar to those reported recently by Mura *et al*. [13] for the interaction of bovine and human strains of *Map *with *A. polyphaga*. Mura *et al*. [13] enumerated the number of *Map *cells in harvested amoebae by quantitative real-time PCR and reported that between 2.5 and 11% of spiked *Map *populations were ingested by *A. polyphaga*. In the present study the number of *Map *within harvested amoebae was quantified by using a published formula [14] which relates BACTEC growth index readings and detection time to numbers of viable *Map *present, yet the ingestion figures obtained were within the same range as Mura *et al*. [13] at between 4.6 and 9.1% for both *Acanthamoeba *spp. Mura *et al*. [13] suggested that perhaps amoebae may only be able to take the mycobacteria up as single organisms and not as clumps. Figure 1(a) shows many *Map *cells concentrated within the vacuole of *A. castellanii *after 3 hours in co-culture at 25°C. Unfortunately, whether these were taken up as individual cells or as clumps of cells cannot be determined. Interestingly, auramine-rhodamine stained smears of 24 month old co-cultures of *A. polyphaga *fed with extracts of human Crohn's disease tissue examined by Mura *et al*. [13] revealed *Map *present throughout the cytoplasm of the amoeba (i.e. not just confined to the vacuole as in this study) or where the amoebae had undergone encystment *Map *were peripherally located.

In this study *Map *clearly demonstrated an ability to survive ingestion by both *Acanthamoeba *spp., and an increase in *Map *numbers occurred after an initial 'lag' period of around 7 d when co-culture was followed for a period of 24 d (Figure 2). This is also in accord with the findings of Mura *et al*. [13] who observed a similar length 'lag' period (8 d) before multiplication (approx. 1 log_10 _increase in numbers) of *Map *within *A. polyphaga *between 8 and 12 days. Since *Map *is a very slow-growing bacterium it is perhaps not surprising that it required an extended period of adaptation to its new intracellular environment before replication commenced. What is unexpected, however, is how *Map *multiplied at all in the absence of adventitious mycobactin. Replication of the closely-related *M. avium *following phagocytosis by *A. castellanii *at temperatures as low as 24°C has previously been reported [10], so it is not unexpected that *Map *behaves in a similar fashion to *M. avium *and, indeed, other mycobacteria studied previously [15]. The observation of the persistence of both *M. avium *and *Map *in the outer walls of the double-walled cysts of *A. polyphaga *[13,16] indicates that *Map *can also survive encystment which would further prolong its survival in extreme environmental conditions. The above studies provide clear evidence of interaction between *Map *and protozoa at ambient laboratory temperatures (20–25°C). Numerous invertebrate and protozoal species have been shown to be present in surface water in Australia [17] so there is certainly scope for such interaction to take place. However, further research will be required to confirm whether interaction occurs between *Map *and protozoa at lower temperatures that may be more representative of natural environmental conditions, certainly in temperate climates.

The bactericidal effect of chlorine on free *Map *in water was studied previously in our laboratory [18]. Log_10 _reductions in the range 1.32–2.82 were observed for *Map *strains NCTC 8578 (bovine) and ATCC 43015 (human isolate) during that study when the same chlorine concentrations and contact times were employed as in the present study. Log_10 _reductions observed for free *Map *NCTC 8578 during the present study fell broadly within the same range (0.78–1.73 log_10_). Log_10 _reductions achieved with ingested *Map *were much lower (0.16–0.94 log_10_) indicating enhanced chlorine resistance when *Map *was taken up by *A. polyphaga*. The elevated chlorine resistance of ingested *Map *is perhaps not unexpected, given that this has previously been observed for coliforms and other bacterial pathogens within protozoa [19]. Enhanced resistance of *Map *to other chemical stressors when taken up by amoebae is also a possibility given that co-culture of *M. avium *with *A. castellanii *reduced the effectiveness of the antimicrobials rifabutin, azithromycin and clarithromycin [20].

## Conclusion

The demonstrated ability of *Map *to survive ingestion by *Acanthamoeba *spp. in the laboratory situation and subsequently multiply clearly illustrates a potential survival and dissemination strategy for this animal pathogen, which may also potentially be implicated in human disease, to use in the natural environment. The enhanced chlorine resistance when ingested by protozoa has clear implications with regard to ensuring the efficacy of chlorination processes during water treatment in order to minimise exposure of the public to this potential pathogen.

## Methods

### Culture preparation

Axenic cultures of *Acanthamoeba castellanii *CCAP 1501/1B and *A. polyphaga *CCAP 1501/3B were maintained in tissue culture flasks containing 10 ml proteose peptone glucose (PPG) medium incubated at 25°C and passed every 4–5 days (volume of passage 1:10). The flasks were incubated flat for optimum growth of the amoebae. The PPG medium consisted of proteose peptone (15 g/l; Oxoid Unipath, Basingstoke, Hampshire, UK) and glucose (18 g/l; Sigma Ltd., Gillingham, UK). The PPG medium was made to volume with amoeba saline solution (ASS) instead of water. The ASS consisted of two stock solutions viz. solution 1, NaCl 24 g/l, MgSO_4_·7H_2_O 0.8 g/l, CaCl_2_·6H_2_O 1.2 g/l; solution 2, Na_2_HPO_4 _28.4 g/l, KH_2_PO_4 _27.2 g/l. The final ASS consisted of 5 ml each of stock solutions 1 and 2 and the mixture made to volume (1 litre) using glass-distilled water.

The three *Map *strains used in this study – NCTC 8578, B2 and ATCC 19698 – had been maintained on Protect cryobeads (Technical Service Consultants, Heywood, UK) at -80°C in order to preserve genetic integrity since entering our laboratory, and had not been repeatedly sub-cultured. For each experiment a working *Map *culture was generated by inoculating a cryobead into Middlebrook 7H9 broth with OADC supplement (oleic acid, albumin, dextrose, catalase; Becton Dickinson, Oxford, UK) and 2 μg/ml mycobactin J (Synbiotics Europe SAS, Lyon, France) and incubating for 6–7 weeks at 37°C.

### Ingestion capacity experiment

*Acanthamoeba castellanii *and *A. polyphaga *were grown in PPG at 25°C for 4 days after which the medium was poured off gently so as not to disturb the bottom surface-attached protozoa. The monolayers were washed gently with ASS once and the monolayer re-suspended in fresh ASS before being incubated overnight at 25°C to starve the protozoa. A working culture of *Map *was centrifuged at 2500 g for 20 min, the pellet washed twice with ASS and finally re-suspended in ASS to give a final cell concentration of approximately 10^7 ^CFU/ml as determined by nephelometry and also subsequent culture in BACTEC 12B medium (Becton Dickinson) supplemented with 2 μg/ml mycobactin J. The number of *Map *incubated with the *Acanthamoeba *spp. was derived from the BACTEC growth index readings using the published formula of Lambrecht et al. [14]. The starved *A. castellanii *and *A. polyphaga *cultures were resuspended by tapping the flasks and the resulting cell suspensions counted using a haemocytometer. The respective protozoan cultures were centrifuged at 400 g for 5 min and the pellets re-suspended with the *Map*-ASS suspensions (10 ml) at a multiplicity of infection (MOI) of 10 *Map *to 1 *Acanthamoeba *cell and incubated at 25°C for 180 min. Following incubation the *Map*-ASS suspension was decanted off and the monolayer washed twice with 10 ml ASS in order to minimise carryover of free *Map*. The protozoan monolayers, after the final washing, were resuspended in 10 ml phosphate-buffered saline supplemented with 1% v/v Tween 80 (PBS-T, pH 7.4; Sigma, Poole, UK) and incubated at 25°C for 10 min before being sonicated (2 × 30 s at 50% intensity with a 10 s interval between bursts) to lyse the amoebae. The lysate was centrifuged at 2,500 g for 20 min and the pellet re-suspended in 10 ml PBS-T and decimally diluted. The dilutions (0.5 ml of each) were inoculated into BACTEC 12B medium, incubated at 37°C and read regularly on a BACTEC 460 machine. Intracellular *Map *counts were derived from the BACTEC growth index readings using the published formula of Lambrecht et al. [14]. This method of quantifying *Map *has been employed in published studies assessing acid resistance [21] and growth of sheep strains of *Map *[22]. Quantitative data derived using this equation must be regarded as approximate since *Map *cells in different physiological states may exhibit different growth responses in the BACTEC culture system. Three replicate runs of this experiment were performed with three strains of *Map*.

### Visualisation of ingested *Map *by acid-fast microscopy

A 10 μl sample of the protozoa/*Map *co-culture was spotted onto a glass slide and dried for 5 minutes at 70°C. The slide was flooded with basic carbol fuschin stain for 15 minutes. The slide was rinsed and flooded with 3 % acid-alcohol (3.0 ml concentrated hydrochloric acid (technical grade) in 97 ml of 95% v/v ethanol) for 1 min. The slide was rinsed and flooded with a 1.0 mg/ml solution of methylene blue for 1 min, which allowed the margins and sub-cellular structure of the amoebae to be visually distinguished. The slide was examined using a Zeiss Axiophot compound microscope under an objective lens (×100).

### Survival of ingested *Map*

*Acanthamoeba *and *Map *NCTC 8578 co-cultures in PPG medium were prepared as described above and incubated at 25°C for up to 24 days. Both *Acanthamoeba *spp. were studied but only one *Map *strain. After 7, 14 and 21 days the PPG medium was replaced to prevent the amoeba encysting [18]. Sub-samples (1 ml) of co-culture were removed after 3 h (0), 2, 4, 7, 14 and 24 days, centrifuged at 2,500 g for 20 min and the pellet re-suspended in PBS-T (10 ml). The suspension was lysed (as described above) and the lysate decimally diluted and inoculated into BACTEC 12B medium and growth monitored and *Map *counts derived after each incubation time as before. The means of three replicate runs were compared by ANOVA.

### Comparative resistance of intracellular and extracellular *Map *to chlorine

A chlorine stock solution (1:1000 dilution) was prepared using Chloros (approx. 10% available chlorine) using distilled reverse osmosis (DRO) water. This was used to prepare test chlorine solutions containing 0, 0.5, 1.0 and 2.0 μg/ml free chlorine. The free chlorine concentrations were confirmed using the N, N-diethyl-p-phenylenediamine ferrous titrimetric method [23]. The test solutions were freshly prepared before each experimental run and were held at room temperature for no longer than 5 min before inoculation. A starved co-culture of *Map *and *A. polyphaga *was prepared as before (incubated for 180 min) along with individual control cultures of *Map *and *A. polyphaga *alone. The prepared test chlorine solutions (10 ml) were inoculated with co-culture and control cultures and incubated at 20°C for the respective contact times of 15 and 30 min. After the appropriate contact time had elapsed the chlorine was neutralised with 1 ml of 1% w/v sodium thiosulphate before the sample was centrifuged at 2500 g for 20 min and the pellet resuspended in 1 ml PBS-T. At this point samples originally inoculated with a co-culture of amoebae and *Map *(i.e. ingested *Map*) were lysed by sonication (as described for ingestion capacity experiment) before dilution, whereas samples inoculated with *Map *only (i.e. free *Map*) were diluted without sonication. Appropriate decimal dilutions were prepared in Maximum Recovery Diluent (Oxoid) and 500 μl of each dilution was inoculated into BACTEC 12B medium, incubated at 37°C, read regularly as before and *Map *counts derived using the published formula [14]. The differences in resistance between amoeba-ingested and free *Map *at varying chlorine concentrations and contact times were compared using ANOVA.

## Authors' contributions

LW carried out the laboratory experiments, acquired and analysed the data, and drafted the manuscript. IG provided training to LW in the culture of *Map *and critically revised the manuscript. MR conceived of the study, participated in its design and coordination and helped to draft the manuscript. All authors read and approved the final manuscript.

## Figures and Tables

**Table 1:** Impact of chlorine concentration (0.5, 1 and 2 μg/ml) and contact time (15 and 30 min) on inactivation of free and *A. polyphaga*-ingested *Map *NCTC 8578 in water.

Chlorine concentration (μg/ml)	15 min contact time		30 min contact time	
	
	Free *Map*	Ingested *Map*	Free *Map*	Ingested *Map*
	Log_10 _reduction (mean ± s.d.)			
0.5	1.27 ± 0.75	0.22 ± 0.25	0.78 ± 0.65	0.16 ± 0.04
1	1.45 ± 0.98	0.51 ± 0.17	1.72 ± 1.05	0.63 ± 0.53
2	1.43 ± 1.24	0.32 ± 0.45	1.73 ± 1.18	0.94 ± 0.56
